# Overexpression of HER-2 in MDA-MB-435/LCC6 Tumours is Associated with Higher Metabolic Activity and Lower Energy Stress

**DOI:** 10.1038/srep18537

**Published:** 2016-01-04

**Authors:** Wieslawa H. Dragowska, Mihaela Ginj, Piotr Kozlowski, Andrew Yung, Thomas J. Ruth, Michael J. Adam, Vesna Sossi, Marcel B. Bally, Donald T. T. Yapp

**Affiliations:** 1 The Department of Experimental Therapeutics, The BC Cancer Agency, 675 West 10th Avenue, Vancouver, BC, Canada V5Z 1L3; 2 The Joint Department of Medical Imaging, University Health Network, 200 Elizabeth St., Toronto, Ont Canada M5G 2C4; 3 The High Field MRI Centre at UBC, University of British Columbia, 2221 Wesbrook Mall, Vancouver, BC Canada V6T 2B5; 4 The TRI-University Meson Facility (TRIUMF), 4004 Wesbrook Mall, Vancouver, BC Canada V6T 2A3; 5 The Faculty of Physics and Astronomy, University of British Columbia, 6224 Agricultural Road, Vancouver, BC Canada V6T 1Z1; 6 The Faculty of Pathology and Laboratory Sciences, University of British Columbia, 2211 Wesbrook Mall, Vancouver, BC Canada V6T 2B5; 7 The Faculty of Pharmaceutical Sciences, University of British Columbia, 2405 Wesbrook Mall, Vancouver, BC Canada V6T 1Z3

## Abstract

Overexpresssion of HER-2 in the MDA-MB-435/LCC6 (LCC6^HER-2^) tumour model is associated with significantly increased hypoxia and reduced necrosis compared to isogenic control tumours (LCC6^Vector^); this difference was not related to tumour size or changes in vascular architecture. To further evaluate factors responsible for HER-2-associated changes in the tumour microenvironment, small animal magnetic resonance imaging (MRI) and positron emission tomography (PET) were used to measure tumour tissue perfusion and metabolism, respectively. The imaging data was further corroborated by analysis of molecular markers pertaining to energy homeostasis, and measurements of hypoxia and glucose consumption. The results showed a strong trend towards higher perfusion rates (~58% greater, *p* = 0.14), and significantly higher glucose uptake in LCC6^HER-2^ (~2-fold greater; *p* = 0.025), relative to control tumours. The expression of proteins related to energy stress (P-AMPK, P-ACC) and glucose transporters (GLUT1) were lower in LCC6^HER-2^ tumours (~2- and ~4-fold, respectively). The *in vitro* analysis showed that LCC6^HER-2^ cells become more hypoxic in 1% oxygen and utilise significantly more glucose in normoxia compared to LCC6^Vector^cells (p < 0.005). Amalgamation of all the data points suggests a novel metabolic adaptation driven by HER-2 overexpression where higher oxygen and glucose metabolic rates produce rich energy supply but also a more hypoxic tumour mass.

Amplification of the HER-2 gene (*c-erb*B2 or *neu* gene) is associated with poor prognosis^1^,^2^,^3^,^4^,^5^,^6^,^7^ and is a therapeutic target of interest in various cancers^3^,^7^,^8^. HER-2 signalling is known to trigger pathways that increase cell proliferation and enhance cell survival, culminating in resistance to anti-cancer therapies^2^,^3^. Our group showed that overexpressing HER-2 in the aggressive MDA-MB-435/LCC6 tumour model^9^ (LCC6^HER-2^) increased the tumour’s viability and levels of hypoxia compared to isogenic control tumours transfected with vector alone (LCC6^Vector^)^10^. High levels of hypoxia would reduce the effectiveness of radiation treatment and chemotherapy, but more importantly is associated with an increased chance of metastasis and lower survival rates^11^,^12^. We now report that LCC6^HER-2^ cells consume more oxygen than their non-HER-2 counterparts which may explain the observed increase in hypoxic cell fraction found in LCC6^HER-2^ tumours (relative to control LCC6^Vector^ tumours). Hypoxia in LCC6^HER-2^ tumours is present concomitantly with increased glucose consumption, suggesting higher rates of glycolysis. The data also indicate that HER-2 expression is associated with improved cell bioenergetics which may provide a survival advantage for HER-2 overexpressing tumours.

## Methods

### LCC6 cells, Tumours and *In Vitro* Oxygen and Glucose Consumption

The identity of the LCC6 parental cell line, MDA-MB-435, was determined to be of melanoma origin rather than breast following a period of controversy and confusion^13^,^14^. Although MDA-MB-435 cells may have been misidentified as breast cancer cells in studies examining the effects of HER-2 overexpression in tumour biology, they are still useful models of altered gene expression^15^,^16^. In our laboratory, the accessibility of MDA-MB-435/LCC6 (LCC6) transfected variants and its consistent tumour take rates in animals provided an opportunity to study the *in vivo* effects of HER-2 overexpression (LCC6^HER-2^) against an isogenic vector transfected tumours (LCC6^Vector^)^10^,^17^.

Animal study and imaging protocols were reviewed and approved by the animal care committee at the University of British Columbia working under the auspices of the Canadian Animal Care Council. All animal procedures and monitoring were carried out in accordance with the approved protocols. Tumours were grown subcutaneously to 200 mm^3^ in immuno-compromised female Rag2M mice^10^ and size matched LCC6^HER-2^ and LCC6^Vector^ tumours were imaged with magnetic resonance imaging (MRI) and positron emission tomography (PET) to evaluate *in situ* tumour tissue perfusion and glucose uptake rates, respectively. Tumours were harvested after imaging and processed for Western Blotting. *In vitro* glucose consumption rates were measured using Amplex® Red Galactose/Galactose Oxidase Assay (Invitrogen); cells were seeded in 6-well plates (5 × 10^5^ cells/3 mL media; DMEM supplemented with 10% FBS (DMEM 10%FBS), Stemcell Technologies, Vancouver, BC) and allowed to adhere overnight, washed twice with glucose free DMEM media, then incubated in DMEM 10%FBS containing 6000, 1000, 500 or 200 μM glucose. The remaining glucose concentrations at 0, 6 and 24 hours were determined and expressed as a percentage of the initial glucose concentrations. The hypoxia marker EF5, which is reduced in the absence of oxygen and forms covalent cellular adducts^18^, was used to evaluate oxygen consumption in a sealed system. Cells (2, 4 and 6 × 10^6^/mL) were suspended in EF5-containing DMEM 10% FBS media (200 μM) and incubated in air-tight tubes for two hours, and after staining with anti-EF5 antibody analysed with flow cytometry, as previously described^10^. The fluorescence intensity of the samples is proportional to the average number of EF5 adducts present in the cell population and is a surrogate measure of oxygen depletion under these conditions.

### Magnetic Resonance Imaging (MRI)

*In situ* tumour perfusion rates were measured using an animal MRI scanner (Bruker, Germany) equipped with a 7T/30 cm horizontal bore magnet. Tumour perfusion measurements were carried out as described previously^19^. Animals were anaesthetized with isofluorane and positioned in a specially designed holder constructed in-house. The animal’s body temperature was maintained at physiological levels, and respiratory rates at ~100 breaths/min during the scan. A catheter was inserted in a tail vein for remote injection of the contrast reagent, Gd-DTPA. A 3 turn solenoid coil surrounding the tumour was used for spin excitation and signal reception. The volume transfer constant (K_trans_) between blood plasma the extra-vascular space in the tumour was calculated from the rapid acquisition of T1-weighted sequences, which monitor the passage of Gd-DTPA in viable tissue, using a two-compartment pharmacokinetic model^20^. When vessels are highly permeable, as in tumours, K_trans_ values approximate blood flow per unit volume of tissue.

### Positron Emission Tomography (PET)

The Concorde-CTI microPET^®^Focus^™^ 120 scanner^21^ was used with ^18^F-fluorodeoxyglucose (FDG), a fluorinated analogue of glucose to evaluate glucose uptake in LCC6^Vector^ and LCC6^HER-2^ tumours. FDG is taken up by cellular glucose transporters and phosphorylated to FDG-6-phosphate and trapped in the cytoplasm^22^,^23^. Regions of interest (ROI) were only drawn on areas of FDG uptake in the PET images to exclude necrotic tissue, and the mean activity from three adjacent planes normalized to the ROI area (mm^2^). Paired sets of mice (5 sets) bearing LCC6^Vector^ and LCC6^HER-2^ tumours were injected intravenously with FDG and scanned simultaneously after 60 minutes.

### Western Blotting

Tumour lysates were analysed by Western blotting as described previously^10^ using antibodies for HER-2, P-AMPK^Thr172^ and P-ACC^Ser79^ (Cell Signaling Technology) and GLUT1 (Chemicon Int.). Protein expression was reported relative to β-actin in the same lane. Western blot analysis was repeated at least twice for each protein.

### Statistics

Statistical analyses were performed with STATISTICA software. One way ANOVA analysis of variance was used to calculate p values. Differences were considered significant at *p* ≤ 0.05. Images of the original Western blots are shown in the supplementary material.

## Results and Discussion

**Figure 1:**
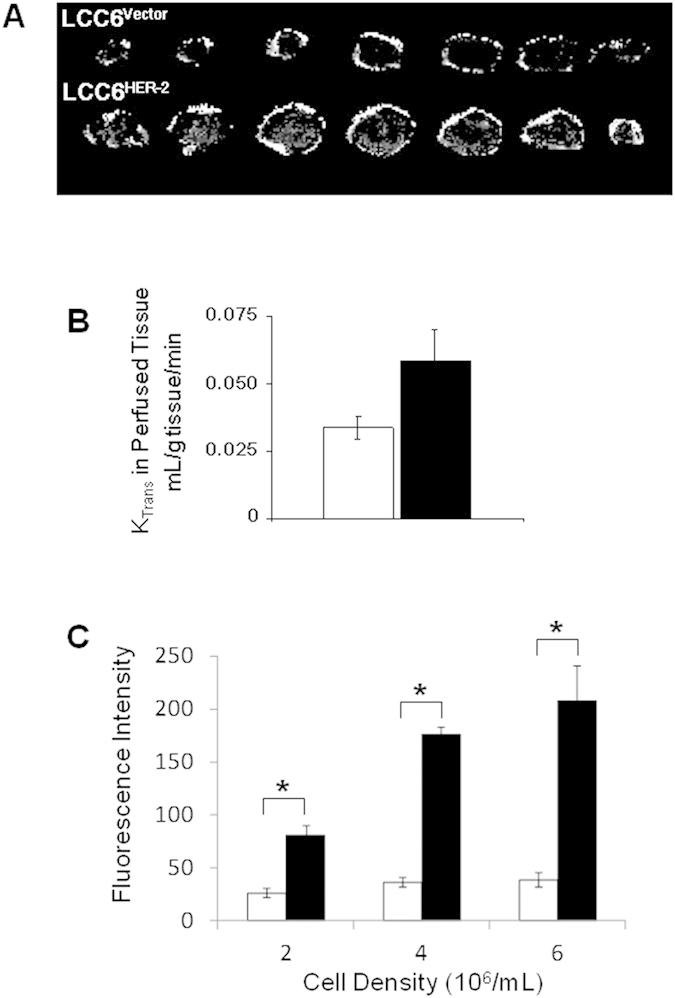
Tissue perfusion rates are higher in LCC6^HER-2^ tumours relative to LCC6^Vector^ tumours. (**A**) Tumour perfusion maps of Gd-DTPA concentrations in edge to edge axial slices of LCC6^Vector^ and LCC6^HER-2^ tumours (top and bottom row, respectively). The bright pixels within the images show areas of tumours which are actively perfused; the intensities correspond to Gd-DTPA concentrations. The areas of perfused tissue are more evenly distributed in LCC6^HER-2^ tumours compared to LCC6^Vector^ tumours. (**B**) K_trans_ values show a strong trend towards higher perfusion rates in LCC6^HER-2^(■) tumours compared to LCC6^Vector^ (□) tumours (median ± SD, *p* = 0.14, n = 4). (**C**) LCC6^Vector^ (□) and LCC6^HER-2^ (■) cells were incubated in air-tight containers containing 1% oxygen in the presence of the hypoxia marker EF5 for two hours followed by staining with anti-EF5 antibody and flow cytometric analysis; fluorescence intensity (arbitrary units) indicates the relative amount of EF5 adducts present in the cells (mean ± SD). The graph shows that LCC6^HER-2^ cells have significantly more adducts than LCC6^Vector^ cells indicating that under these conditions, they consume more oxygen and become hypoxic faster. Significant differences (*p* < 0.001) are indicated by the asterisk (*).

Previously, we showed that HER-2 overexpression in the LCC6 (LCC6^HER-2^) tumour model increases tissue hypoxia and overall tumour viability without changing vascular parameters; i.e., vessel density, numbers of functional Hoechst 33342 perfused vessels and the median distance of tissue to blood vessels (MDV)^10^. The classical model of tumour hypoxia is based on an oxygen gradient that emanates outwards from the blood vessel to tissue^24^; thus an increase in hypoxia without changes in the vascular parameters would imply that factors other than oxygen delivery are at play. A possible reason for this could be impaired tissue perfusion; K_trans_, a surrogate marker of tissue perfusion was thus measured with MRI. The MR images show that LCC6^HER-2^ tumours have more areas of active blood flow (bright pixels) than LCC6^Vector^ controls (Fig. 1A). The K_trans_ values reported here represent the relative blood flow rates in tumour tissue. The median K_trans_ value in LCC6^HER-2^ tumours was on average 58% greater than LCC6^Vector^ tumours (0.059 ± 0.011 and 0.034 ± 0.0041 (n = 4) ml/g tissue/min, respectively) although the difference was not statistically significant (*p* = 0.14, Fig. 1B). However, these results strongly support the notion that the emergence of hypoxia in LCC6^HER-2^ tumours is not due to poor blood flow and suggest that oxygen and nutrients may be supplied equally and diffuse the same distance in both tumours. In fact, given the strong trend to higher perfusion in LCC6^HER-2^ tumours, a decrease in hypoxia might be expected.

LCC6^HER-2^ overexpressing tumours could also develop more hypoxia than controls because of differences in oxygen metabolism. This alternative interpretation is corroborated by the *in vitro* data showing that LCC6^HER-2^, compared to the LCC6^Vector^ cells, become more hypoxic in a 1% oxygen environment (Fig. 1C; *p* < 0.001). The hypoxia marker EF5 is used here as a surrogate measure of oxygen depletion in the closed system. The data show that HER-2 overexpressing cells have higher levels of EF5 staining suggesting they consume more oxygen than the vector controls. In turn, higher oxygen consumption reflects increased rates of oxidative processes (e.g. oxidative metabolism of energy substrates such as glucose or fatty acids) for producing ATP. In a tumour, the demand for oxygen by tumour cells could outstrip the supply and create areas of hypoxia even in vascularized tissue.

Increased hypoxia associated with HER-2 overexpression is not unique to the LCC6 model; overexpressing HER-2 in the MCF7 breast cancer tumour model resulted in higher hypoxia levels as shown with pimonidazole staining of tumour sections, and lower tissue oxygenation measured directly using an intratumoural pO_2_ probe^25^. Moreover, as in our study, vascular parameters including MDV were similar in HER-2 overexpressing and control MCF7 tumours, and inhibiting HER-2 with trastuzumab resulted in better oxygenation^25^. However, tumour tissue perfusion rates were not measured in this study so the status of oxygen delivery in these tumours is unknown. Interestingly, recent studies reported that the transcription factor NF-κB is an important physiological regulator of mitochondrial respiration^26^. The authors suggested that increased mitochondrial respiration is a metabolic adaptation to maintain energy homeostasis in growing cells and tumours under conditions where nutrients are in high demand to meet cellular needs of proliferating cells. As previously shown^10^, LCC6^HER-2^ cells and tumours express NF-κB at much higher levels than LCC6^Vector^ controls which may account for higher oxidative metabolism in HER-2 overexpressing LCC6 tumours.

In contrast to our results showing the association of HER-2 expression with increased oxygen consumption (Fig. 1C), studies from other groups showed that expression of HER-2 in various cell lines promotes a metabolic shift from mitochondrial respiration (oxidative metabolism) to glycolysis *via* intermediary molecules^15^,^16^,^27^,^28^, and the data suggested that HER-2 overexpression was associated with lower oxygen consumption compared to controls^15^,^16^,^27^,^28^. The different methods of measuring oxygen consumption, culture conditions, cell type specific pathways regulating metabolism *in vitro*, and potential interference from molecular silencing of metabolic pathways used in these studies, however, make a direct comparison between these results^15^,^16^,^27^,^28^ and ours difficult. Moreover, the *in situ* oxygenation or hypoxia levels in tumours were not measured in these reports so it is difficult to conclude whether the observed *in vitro* differences due to HER-2 activity^15^,^16^,^27^ would be present *in vivo* as well.

**Figure 2:**
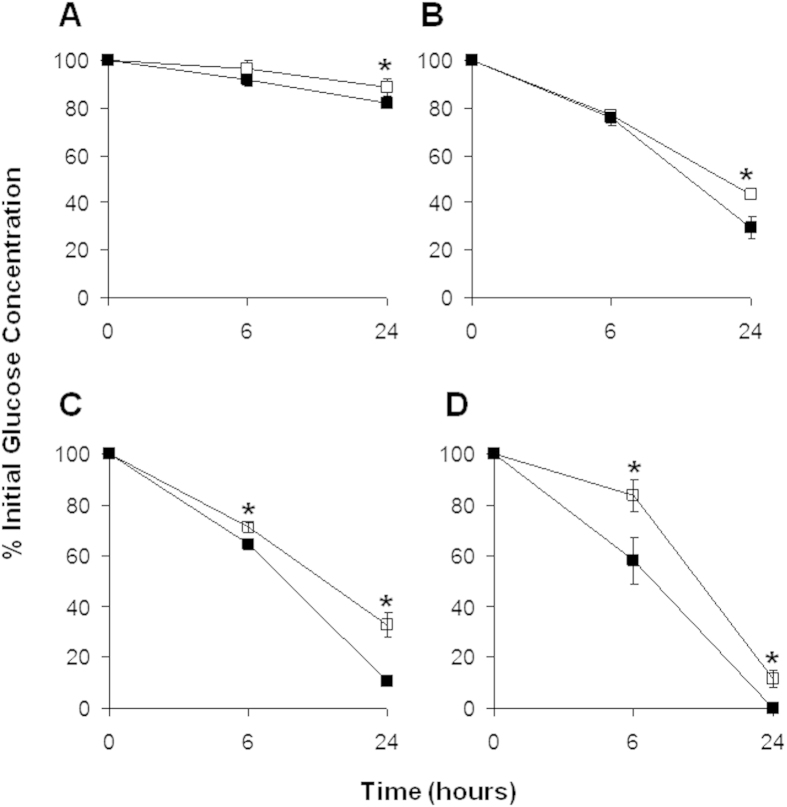
LCC6^HER-2^ cells consume more glucose than LCC6^Vector^ cells. Graphs (**A–D**) show the consumption of glucose by LCC6^Vector^ (□) and LCC6^HER-2^ (■) cells over a 24 hour period (results expressed as the percentage of glucose remaining in the media). The rates of glucose consumption are dependent on the glucose present in the media:at 6000 and 1000 μM glucose (A and B, respectively) differences in consumption rates become significant after 24 hours; however at 500 and 200 μM glucose (C and D, respectively), the differences are apparent by 6 hours. Significant differences (*p* < 0.005) are indicated by the asterisk (*).

To investigate if changes in oxygen consumption associated with HER-2 expression affected glucose metabolism we measured glucose consumption in LCC6^HER-2^ and LCC6^Vector^ cells. Our results showed that LCC6^HER-2^ cells have higher glucose consumption in an aerobic environment (Fig. 2). This finding was consistent with the findings published in the same reports referenced above^15^,^16^,^27^,^28^. Interestingly, in the LCC6 model, the rate of consumption appears to be dependent on the glucose concentration; at 6000 and 1000 μM glucose, significant differences in glucose consumption between LCC6^Vector^ and LCC6^HER-2^ cells were noted at 24 hours (88% vs. 82%; *p* = 0.03 and 43% vs. 29%; *p* = 4.8 × 10^−7^, for 6000 and 1000 μM, respectively), whereas at 500 and 200 μM, significant differences were apparent by 6 hours (71% vs. 64%; *p* = 0.001 and 83% vs. 58%; *p* = 0.001). These data imply that HER-2 overexpressing LCC6 cells may respond faster to changes in its environment, i.e., glucose deprivation.

**Figure 3:**
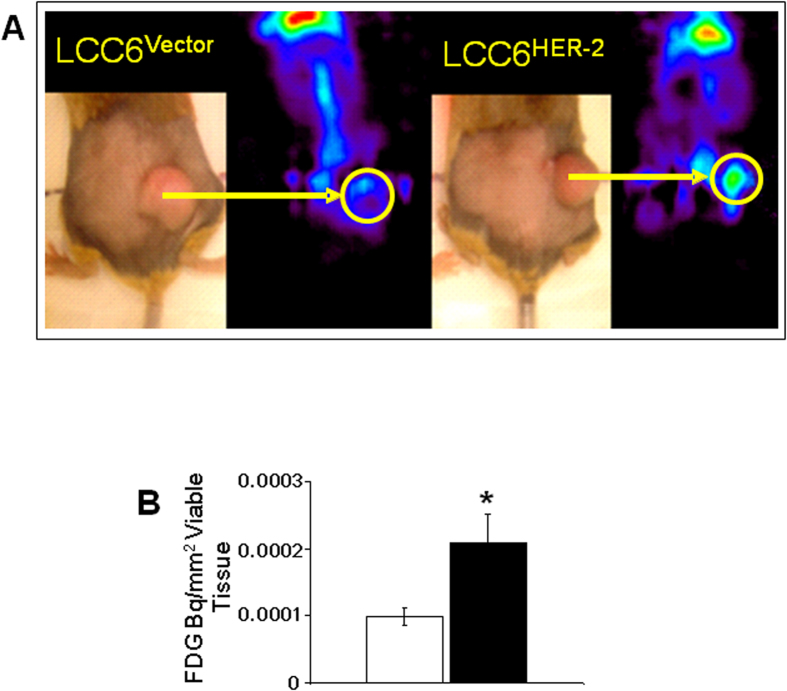
FDG uptake is higher in LCC6^HER-2^ tumours. (**A**) PET images of *in situ* FDG accumulation in LCC6^Vector^ and LCC6^HER-2^ tumours; the tumour is circled in yellow and the intensity of green areas in the tumour reflects the activity of phosphorylated FDG trapped in cells. In the color scale used, purple is low activity, red is high activity. (**B**) Total accumulation of FDG present in regions of interest (viable tissue only) drawn on PET images of LCC6^Vector^ (□) and LCC6^HER-2^ (■) tumours (mean ± SD; *p* = 0.025, n = 5). Significant differences (*p* < 0.05) are indicated by the asterisk (*).

Most importantly, we also confirm higher rates of glucose metabolism *in vivo* by measuring *in situ* metabolic activity in LCC6^HER-2^ and LCC6^Vector^ tumours using PET. The data show that FDG signal in LCC6^HER-2^ is more evenly distributed and more intense than in LCC6^Vector^ tumours (Fig. 3A). The *in situ* accumulation of FDG per unit area of viable tissue show that LCC6^HER-2^ tumours utilize on average ~2-fold more glucose than LCC6^Vector^ tumours (2.09 × 10^-4^ ± 4.26 × 10^−5^ Bq/mm^2^ and 9.89 × 10^−5^ ± 1.4 × 10^−5^ Bq/mm^2^ respectively; *p* = 0.025; n = 5 for each tumour group; Fig. 3B). Malignant tissue is known to use more glucose than normal tissue^29^ due to the Warburg effect^30^. Cells transformed with oncogenes, including HER-2, have also been shown to elevate levels of glucose transporters and glucose consumption *in vitro*, although the molecular pathways involved were not elucidated^31^,^32^,^33^. HER-2 overexpression was also shown to enhance the activation of proteins involved in glycolytic, metabolic, stress-responses and detoxification processes in the breast tumour microenvironment^34^. Others demonstrated a glycolytic shift based on higher levels of lactate dehydrogenase A in tumours derived from cell lines obtained from mammary gland tumours of *neu*-transgenic mice^28^. To the best of our knowledge, we provided the first direct, functional measurements showing increased glucose uptake *in vivo* in the HER-2 background in a set of otherwise isogenic tumours. It is uncertain to what extent this increase is a reflection of a glycolytic shift in the oxygenated tumour tissue or an increased metabolic activity of a hypoxic fraction present in LCC6^HER-2^ tumours. However, considering the results showing increased glucose consumption in aerobic conditions in LCC6^HER-2^ compared to LCC6^Vector^ cells (Fig. 3) it is reasonable to speculate that HER-2 mediates a glycolytic shift in oxygenated tissue in LCC6^HER-2^ tumours.

**Figure 4:**
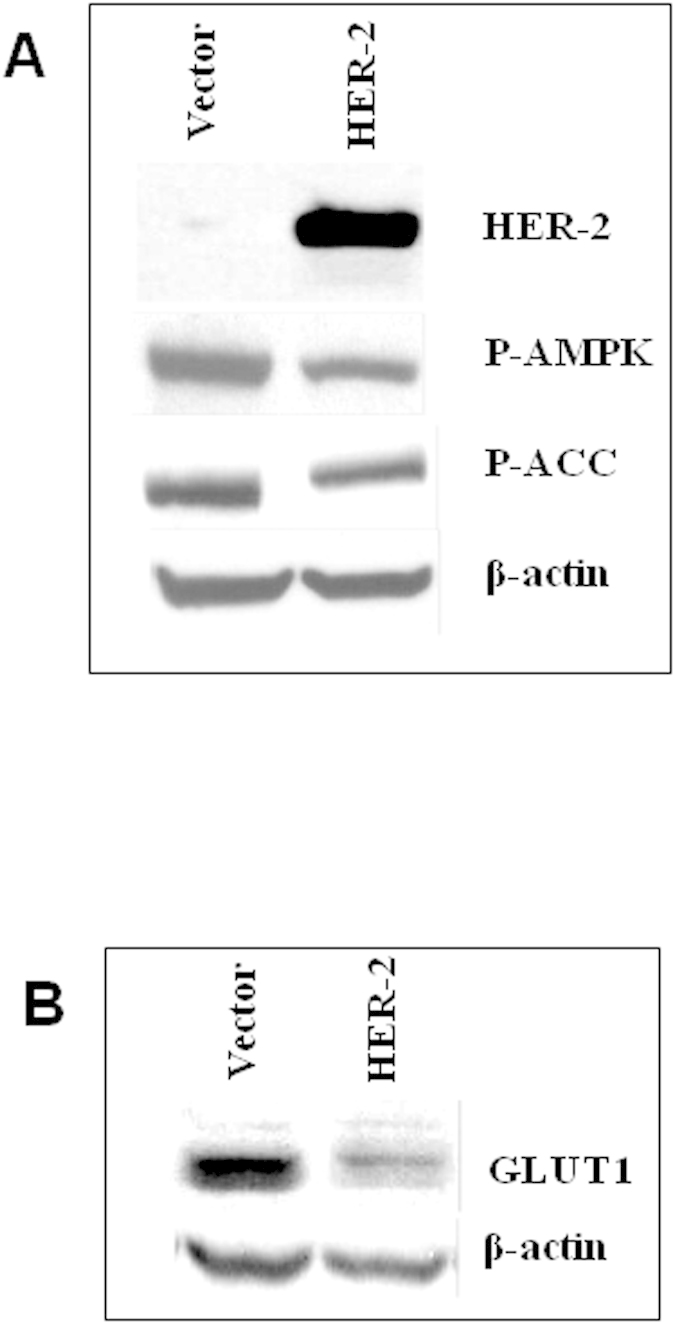
(**A**) Expression levels of P-AMPK, an indicator of energy stress, are lower in LCC6^HER-2^ relative to LCC6^Vector^ tumours; as expected, the expression levels of a downstream effector of P-AMPK, P-ACC, follow the same trend (P-AMPK: phosphorylated AMPK; P-ACC: phosphorylated ACC). (**B**) Western blot analysis shows that LCC6^HER-2^ tumours have lower expression levels of GLUT1, the major transmembrane transporter for glucose, relative to LCC6^Vector^ tumours. Representative results from two tumours are shown in the figure. For clarity, the bands in the figure were cropped from the original gel. Gels were run under the same experimental conditions. The images of the full gels are available in the supplementary material.

Higher oxygen consumption is also a reflection of a greater ATP demand, thus, a parallel increase in oxidative and glycolytic metabolism would appear to be a versatile and advantageous adaptation to balance bioenergetic stress in HER-2 overexpressing tumours to sustain proliferation and survival of cancer cells. In line with this rationale molecular factors germane to cell bioenergetics were examined. Expression levels of phosphorylated-5′-AMP-activated protein kinase (P-AMPK) the activated form of AMPK, an important regulator of energy processes in cells^35^ provide further insight into the bioenergetics of the LCC6 tumours. AMPK is phosphorylated when the ratio of AMP to ATP in the cell is high – i.e. when the concentration of ATP is too low to sustain cell functions and the cell enters a state of energy stress^36^. P-AMPK subsequently activates various pathways that produce energy and minimizes cellular energy expenditure in an overall bid to raise ATP levels^35^,^36^. The analysis of activated AMPK (P-AMPK) showed that expression of this protein was lower (~2-fold) in LCC6^HER-2^ compared to LCC6^Vector^ tumours; levels of phosphorylated Acetyl CoA Carboxylase (P-ACC), a downstream effector of AMPK, also trended lower (Fig. 4A). These data provided further insight into the bioenergetics of the LCC6 tumours and suggested that LCC6^HER-2^ tumours appear to have sufficient ATP to meet the energy demand in contrast to control LCC6^Vector^ tumours.

The lower energy stress in LCC6^HER-2^ tumours was also reflected in greatly reduced (~4-fold) expression of glucose transporter-1 (GLUT1) *in vivo* (Fig. 4B). Since GLUT1 is responsible for transporting glucose (and FDG), across the cell membrane^22^,^23^, this result appears counterintuitive considering that FDG uptake was greater in LCC6^HER-2^ compared to LCC6^Vector^ tumours. However, we argue that low expression levels of P-AMPK in LCC6^HER-2^ tumours indicate that tumours are replete with ATP and thus well-energized; consequently there is no need to upregulate GLUT1 levels in LCC6^HER-2^ tumours. In contrast, existing energy stress in LCC6^Vector^ tumours possibly results in sufficient expression of GLUT1 in order to balance energy needs, as described in other systems^37^. Based on our data it is reasonable to hypothesize that the high uptake of FDG in LCC6^HER-2^ tumours is not due solely to GLUT1 levels, but also to activation of glycolytic enzymes by HER-2^34^ that shuttle glucose into glycolytic pathways at a higher rate^23^,^38^,^39^. The net result is an increase in overall glucose flux through the cell consistent with the higher levels of FDG uptake in LCC6^HER-2^ tumours.

In conclusion, we hypothesize, on the basis of the present data, that overexpression of HER-2 in the LCC6 tumour model is associated with higher rates of oxidative metabolism in addition to increased levels of glycolytic activity. Joining these two functions ultimately produces a more hypoxic tumour, but one that efficiently meets its energy demand. Most importantly, the present data establish a link between HER-2 signalling and cellular metabolic processes which warrants further investigation in other tumour models where HER-2 overexpression is clinically relevant. On-going work in our laboratory is focussed on the putative roles that HER-2 activation play in metabolic processes as in the long-term such studies may identify novel metabolic targets for the treatment of HER-2 positive cancers.

## Additional Information

**How to cite this article**: Dragowska, W. H. *et al.* Overexpression of HER-2 in MDA-MB-435/LCC6 Tumours is Associated with Higher Metabolic Activity and Lower Energy Stress. *Sci. Rep.* **6**, 18537; doi: 10.1038/srep18537 (2016).

## Supplementary Material

Supplementary Information
