# Supplementary material for: Overexpression of HER-2 in MDA-MB-435/LCC6 Tumours is Associated with Higher Metabolic Activity and Lower Energy Stress
Source: Sci Rep. 2016 Jan 4;6:18537. doi: 10.1038/srep18537 (PMC4698760; doi:10.1038/srep18537)
Supplement: Supplementary Information [file srep18537-s1.pdf]

# OVEREXPRESSION OF HER-2 IN MDA-MB-435/LCC6 TUMOURS IS ASSOCIATED WITH HIGHER METABOLIC ACTIVITY AND LOWER ENERGY STRESS.

Wieslawa H. Dragowska, ^Mihaela Ginj, Piotr Kozlowski, Andrew Yung,
Thomas J. Ruth, Michael J. Adam, Vesna Sossi, Marcel B. Bally,
*Donald T.T. Yapp

^Formerly at The Department of Experimental Therapeutics, The BC Cancer Agency

***Corresponding Author**: Donald T.T. Yapp: Experimental Therapeutics, BC Cancer Research Centre, 675 West 10th Ave, Vancouver, British Columbia, Canada V5Z 1L3. (604) 675 8023; dyapp@bccrc.ca

HER-2

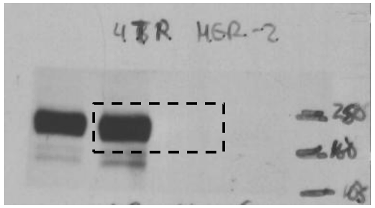

**H2 H1 V2 V1**

Actin

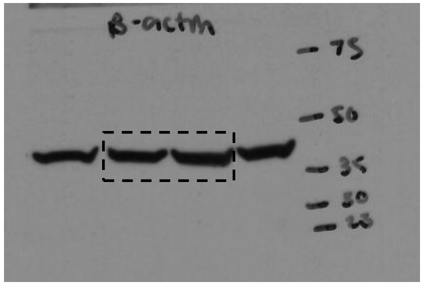

**H2 H1 V2 V1**

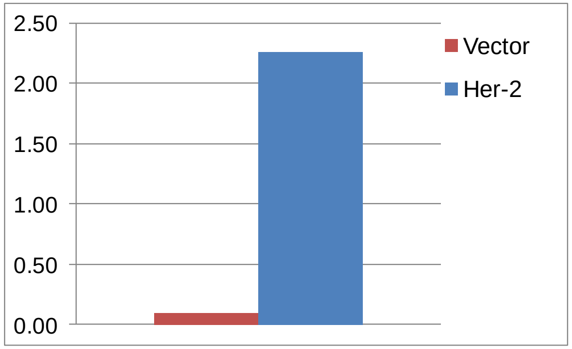


Densitometry ratio of protein bands to actin (n=2)

P-AMPK

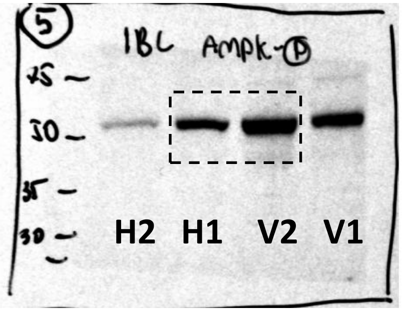


Actin

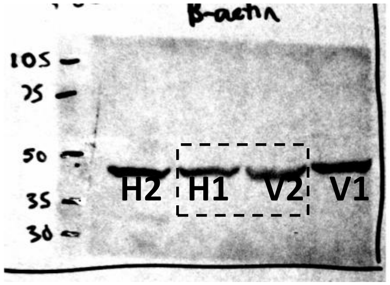


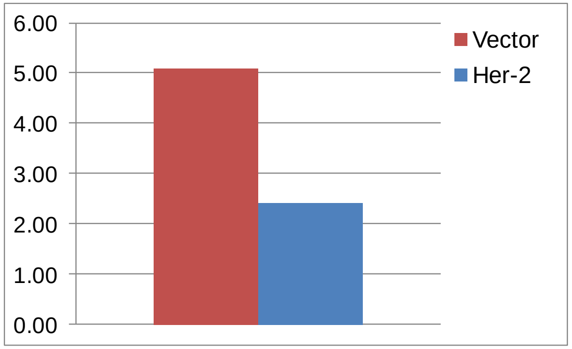


Densitometry ratio of protein bands to actin (n=2)

Key: H2, H1 = LCC6$^{HER-2}$ tumours; V1, V2 = LCC6$^{Vector}$ tumours; dashed lines indicate cropping box.
Note that cropped images were reversed so that Vector and HER-2 tumours are on the left and right, respectively in Figure 4

P-ACC

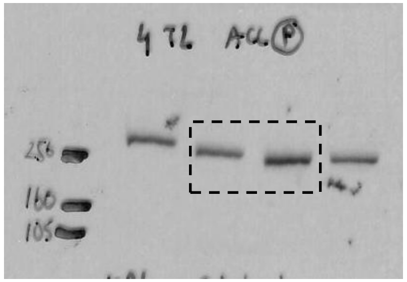

H2 H1 V2 V1

Actin

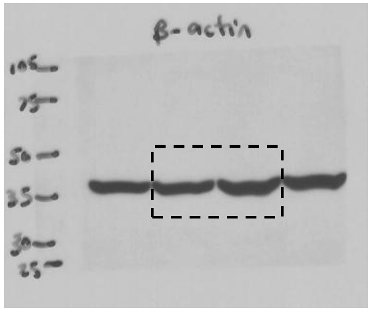

H2 H1 V2 V1

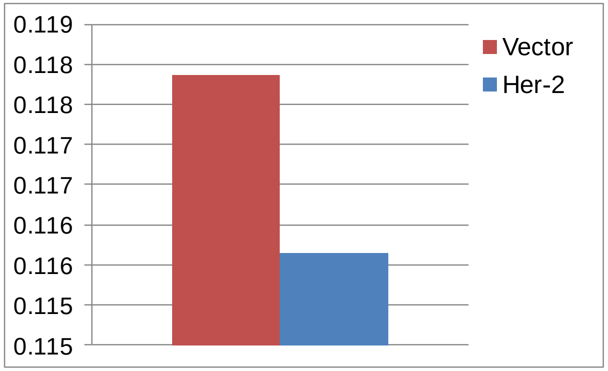


Densitometry ratio of protein bands to actin (n=2)

GLUT-1

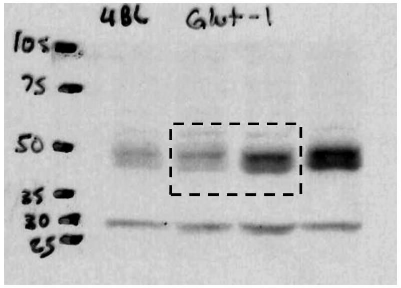

H2 H1 V2 V1

Actin

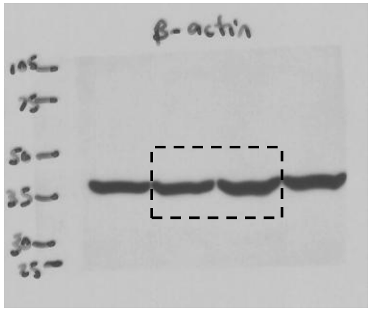

H2 H1 V2 V1

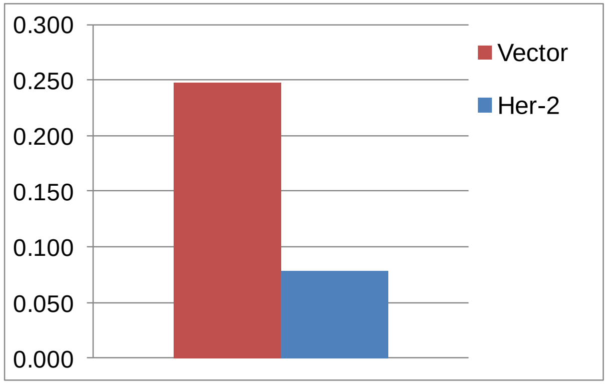


Densitometry ratio of protein bands to actin (n=2)

Key: H2, H1 = $LCC6^{HER-2}$ tumours; V1, V2 = $LCC6^{Vector}$ tumours; dashed lines indicate cropping box.
Note that cropped images were reversed so that Vector and HER-2 tumours are on the left and right, respectively in Figure 4
